# Distribution Indices of Magnetic Susceptibility Values in the Primary Motor Cortex Enable to Classify Patients with Amyotrophic Lateral Sclerosis

**DOI:** 10.3390/brainsci12070942

**Published:** 2022-07-18

**Authors:** Mauro Costagli, Graziella Donatelli, Paolo Cecchi, Paolo Bosco, Gianmichele Migaleddu, Gabriele Siciliano, Mirco Cosottini

**Affiliations:** 1 Department of Neuroscience, Rehabilitation, Ophthalmology, Genetics, Maternal and Child Sciences (DINOGMI), University of Genoa, 16132 Genoa, Italy; mauro.costagli@unige.it; 2 Laboratory of Medical Physics and Magnetic Resonance, IRCCS Stella Maris Foundation, 56128 Pisa, Italy; paolo.bosco@fsm.unipi.it; 3 Imago 7 Foundation, 56128 Pisa, Italy; paolo.cecchi@fsm.unipi.it; 4 Neuroradiology Unit, Azienda Ospedaliero-Universitaria Pisana, 56124 Pisa, Italy; gm.migaleddu@gmail.com (G.M.); mirco.cosottini@unipi.it (M.C.); 5 Neuroradiology Unit, Department of Translational Research on New Technologies in Medicine and Surgery, University of Pisa, 56126 Pisa, Italy; 6 Neurology Unit, Department of Clinical and Experimental Medicine, University of Pisa, 56126 Pisa, Italy; gabriele.siciliano@unipi.it

**Keywords:** Amyotrophic Lateral Sclerosis, Magnetic Resonance Imaging, Quantitative Susceptibility Mapping, motor cortex, radiological biomarker, diagnostic accuracy

## Abstract

Quantitative Susceptibility Mapping (QSM) can measure iron concentration increase in the primary motor cortex (M1) of patients with Amyotrophic Lateral Sclerosis (ALS). However, such alteration is confined to only specific regions interested by upper motor neuron pathology; therefore, mean QSM values in the entire M1 have limited diagnostic accuracy in discriminating between ALS patients and control subjects. This study investigates the diagnostic accuracy of a broader set of M1 QSM distribution indices in classifying ALS patients and controls. Mean, standard deviation, skewness and kurtosis of M1 QSM values were used either individually or as combined predictors in support vector machines. The classification performance was compared to that obtained by the radiological assessment of T2* signal hypo-intensity of M1 in susceptibility-weighted MRI. The least informative index for the classification of ALS patients and controls was the subject’s mean QSM value in M1. The highest diagnostic performance was obtained when all the distribution indices of positive QSM values in M1 were considered, which yielded a diagnostic accuracy of 0.90, with sensitivity = 0.89 and specificity = 1. The radiological assessment of M1 yielded a diagnostic accuracy of 0.79, with sensitivity = 0.76 and specificity = 0.90. The joint evaluation of QSM distribution indices could support the clinical examination in ALS diagnosis and patient monitoring.

## 1. Introduction

The infiltration of iron-rich microglia in the deep layers of the primary motor cortex (M1) [1] is a feature of upper motor neuron pathology in Amyotrophic Lateral Sclerosis (ALS). Such increase in iron concentration has been best appreciated in vivo with Magnetic Resonance Imaging (MRI) by using techniques based on Gradient Recalled Echo (GRE) sequences: images with pronounced T2*-contrast and Susceptibility-Weighted Imaging (SWI) [2] reveal the presence of a hypo-intense rim in M1 [3,4,5,6]. The recently established technique of Quantitative Susceptibility Mapping (QSM) [7] manipulates GRE magnitude and phase images to obtain a quantitative measure of a magnetic property of tissues, namely magnetic susceptibility (χ), which reflects tissue concentrations of paramagnetic (χ > 0, such as iron) and diamagnetic (χ < 0, such as calcium and lipids) compounds in each voxel. The M1 hypo-intensity in SWI and T2*-weighted images translate into increased QSM values [8,9,10,11] associated with the degree of microglial activation [12]. It has been demonstrated that the χ increase in M1 in ALS patients involves only the deep layers of the cortex [1,4,9] and it is confined to only specific regions of the motor homunculus [13] affected by upper motor neuron pathology [14] (e.g., the paracentral lobule, the hand-knob and the orofacial region). Given these premises, a simple analysis of mean QSM values may not reveal statistically significant differences between ALS patients and controls when the entire M1 is considered: indeed, a recent study demonstrated that the skewness and standard deviation of the QSM value distribution within M1, and not the mean value, best capture the differences between ALS patients and controls [15]. 

In this study we aimed to investigate the diagnostic accuracy of distribution indices (mean, standard deviation, skewness and kurtosis) of QSM values in M1, taken either individually or as combined predictors in support vector machines (SVM) which could enable to improve the classification of ALS patients and controls.

## 2. Materials and Methods

This is a retrospective study including the QSM datasets of all subjects who underwent the acquisition sequence described in Table 1 from May 2016 to November 2019 and who satisfied either one of the following criteria:

- Outpatients of the Neurological Unit of Pisa University Hospital with a diagnosis of definite or probable ALS [16] and no concomitant neurodegenerative diseases (51 subjects, mean age 64 ± 10 years, 23 males and 28 females). Spasticity, deep tendon reflexes, clonus, Babinsky sign and pseudobulbar affect were assessed to produce the Upper Motor Neuron (UMN) score [4] of each patient.
- Subjects with negative anamnesis for motor neuron diseases and psychiatric disorders (10 subjects, mean age 58 ± 13 years, 3 males and 7 females).

The Mann–Whitney U-test was used to verify that subjects’ age differences in the two groups were not significant (*p* = 0.15).

All data were acquired at the Neuroradiology Unit of Pisa University Hospital on the same 3T scanner (Discovery MR750, General Electric, Chicago, IL, USA).

Magnitude and phase images acquired with a 3D Gradient Recalled Echo (GRE) multi-echo sequence were processed by following an established pipeline [17] consisting of brain masking [18], phase unwrapping [19], background field removal [20] and magnetic dipole inversion [21] to generate one resultant QSM image [22] for each subject. 

Regions of Interest (ROI) representing M1 were obtained from the right and left Primary Motor Cortex of the Harvard Oxford Cortical Atlas [23]. The co-registration of the M1 ROI to the QSM images of each subject was achieved by concatenating (1) the affine and nonlinear transformations describing the registration between the atlas template and the subject’s T1-weighted anatomical scan and (2) the rigid-body transformation describing the registration between the subject’s T1-weighted anatomical scan and the GRE scan used to generate QSM images, by using FLIRT and FNIRT in FSL [24]. ROIs were visually inspected by one neuroradiologist and, where necessary, they were manually edited in order to include missing parts of M1 and exclude cortical regions that were erroneously included by the automatic pipeline. 

The following distribution indices of QSM values in M1 were considered: mean value, standard deviation, skewness (which reflects the asymmetry of the distribution) and kurtosis (which reflects the amount of outliers). These indices were calculated in two ways: (1) by considering all QSM values in the entire bilateral M1 ROIs of each subject (indicated by μ, σ, S and K, respectively); (2) by considering only the positive QSM values within the ROI (μ+, σ+, S+ and K+) to exclude voxels with predominantly diamagnetic tissue, which are likely to represent subcortical white matter. The differences in the distribution indices between the two groups of subjects were assessed by the Mann–Whitney U-test. A *p*-value, *p* = 0.05, was set as the threshold for statistical significance. The diagnostic accuracy of each of these indices was assessed in terms of the Area Under the Receiver-Operating-Characteristic Curve (AUC). 

To assess the diagnostic accuracy of each individual distribution index and all their possible combinations, $\overset{4}{\underset{c=1}{\sum }}\;\left(\begin{array}{c} 4\\ c \end{array}\right)=15$ different Support Vector Machine (SVM) classifiers [25] were considered for each of the two selection criteria of QSM values (that is, either all QSM voxels in M1 or only the positive QSM voxels). Each classifier was trained 1000 times, each time by using randomly chosen 45 ALS patients and 9 controls and tested on the left-out subjects (6 ALS patients and 1 control). To account for the unbalance between the number of patients and controls in the training phase, classification errors on controls (FP, false positives) were assigned a weight 459=5 times higher with respect to classification errors on ALS patients (FN, false negatives). Each classifier was evaluated on the basis of its diagnostic accuracy $A=\frac{TP+TN}{TP+TN+FP+FN}$, where TP (true positives) represent the number of subjects correctly classified as ALS patients and TN (true negatives) is the number of subjects correctly classified as controls.

Sensitivity and specificity of the SVM classifiers were compared with the classification obtained by radiological assessment, performed by one experienced neuroradiologist, of T2* signal hypo-intensity of M1 in susceptibility-weighted imaging [4].

## 3. Results

The 51 patients with ALS who participated in this study had bulbar onset in 14 cases (27%), upper limb onset in 8 cases (16%) and lower limb onset in 29 cases (57%). With respect to the MR exam, time from symptom onset and time from diagnosis were 13.1 ± 9.2 and 1.2 ± 5.3 (mean ± standard deviation) months, respectively. Their UMN scores were 8.5 ± 5.4.

**Figure 1:**
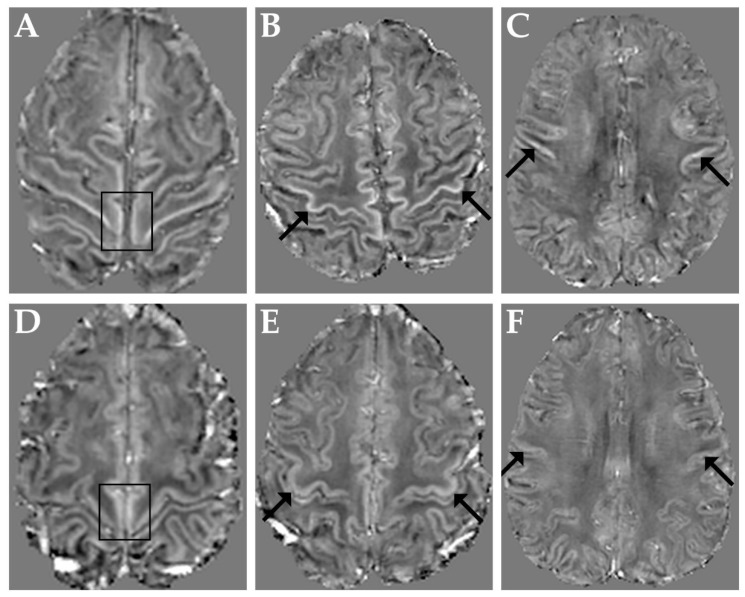
Top row: representative QSM images obtained in three ALS patients with M1 χ increase in the paracentral lobules (**A**), in both hand-knob areas (**B**) and in the orofacial regions (**C**). Bottom row: QSM images in a representative control subject at the level of the paracentral lobules (**D**), hand-knob (**E**) and orofacial regions (**F**). Arrows and boxes indicate corresponding regions in patients and control subject.

Figure 1 shows representative QSM images obtained in three ALS patients with M1 χ increase in the paracentral lobule, in the hand-knob and in the orofacial region (panels A–C, respectively) and, for reference, in a control subject (panels D–F).

**Figure 2:**
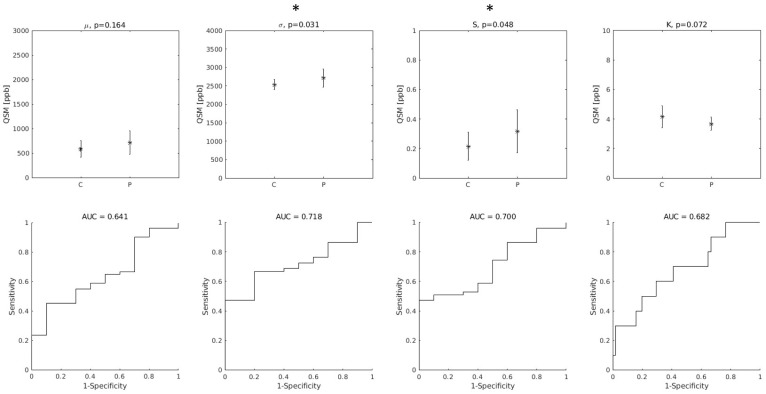
(Top row): group differences between Controls (C) and ALS patients (P) in the distribution indices of M1 QSM values. μ: mean value; σ: standard deviation; S: skewness; K: kurtosis. Asterisks (*) indicate statistically significant differences. (Bottom row): diagnostic accuracy of each feature. AUC: Area Under the Receiver-Operating-Characteristic Curve.

Among the distribution indices of QSM values in the entire bilateral M1, σ and S were those that exhibited statistically significant differences between ALS patients and controls. The AUC were 0.72 and 0.70 for σ and S, respectively. Interestingly, the smallest AUC was obtained with μ, as shown in Figure 2. 

**Figure 3:**
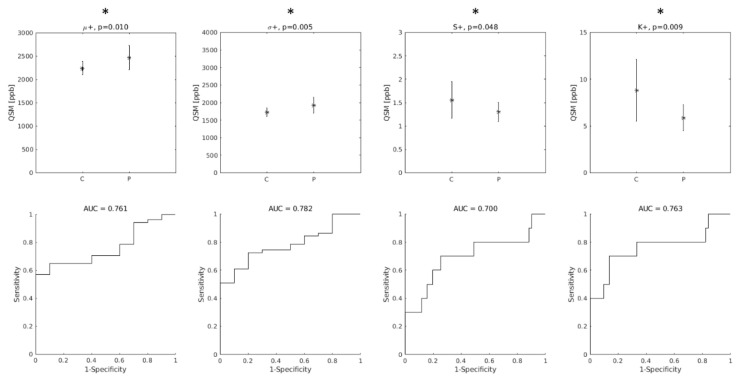
Top row: group differences between Controls (C) and ALS patients (P) in the distribution indices of M1 QSM positive values. μ+: mean value; σ+: standard deviation; S+: skewness; K+: kurtosis. Asterisks indicate statistically significant differences. Bottom row: diagnostic accuracy of each feature. AUC: Area Under the Receiver-Operating-Characteristic Curve.

When only the positive QSM values were considered, the differences between groups were statistically significant for all indices, and the AUC values improved (AUC = 0.76, 0.78, 0.70 and 0.76 for μ+, σ+, S+ and K+, respectively, as shown in Figure 3). 

**Figure 4:**
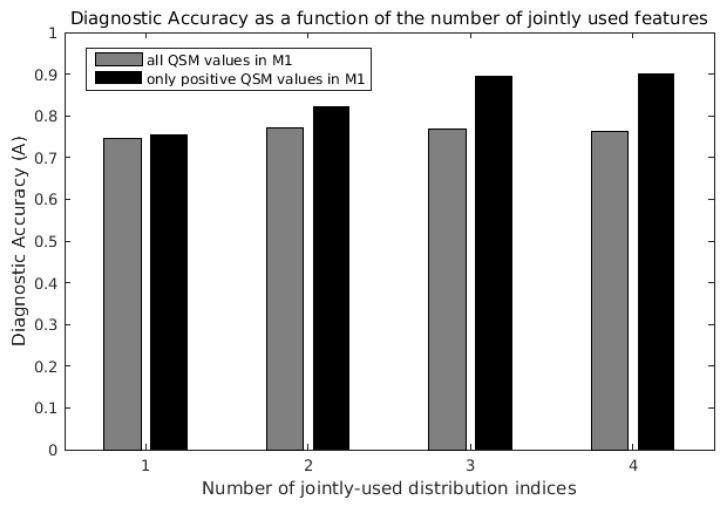
Maximum diagnostic accuracy of support vector machine (SVM) classifiers as a function of the number of QSM distribution indices jointly considered. The last black bar on the right indicates the maximum diagnostic accuracy (A = 0.90) obtained with the joint use of all four distribution indices μ+, σ+, S+ and K+ of QSM positive values in the primary motor cortex (M1).

When QSM distribution indices were used individually in SVM classifiers, the parameter that enabled the best diagnostic accuracy was S+ (A = 0.76, first black bar on the left in Figure 4). The least informative feature was, again, μ (A = 0.43). 

When the QSM distribution indices were jointly used in the SVM classification, the diagnostic accuracy increased, and it was always higher when only the positive QSM values in the ROI were considered (Figure 4, black bars vs. gray bars). The diagnostic accuracy was highest (A = 0.90, fourth black bar on the right in Figure 4) when μ+, σ+, S+ and K+ were jointly used, obtaining sensitivity = 0.89 and specificity = 1. For comparison, the radiological assessment of T2* signal hypo-intensity of M1 in susceptibility-weighted imaging [4] yielded a diagnostic accuracy of 0.79, sensitivity of 0.76 and specificity of 0.90.

Interestingly, in the analysis including all QSM voxels in M1 (light gray bars), the classification performance did not monotonously increase with the number of jointly-used indices: when μ was added to the three-feature set composed by σ, S and K, the diagnostic accuracy slightly decreased from 0.77 (third gray bar) to 0.76 (fourth gray bar).

## 4. Discussion

This study investigated the capability to discriminate between ALS patients and controls on the basis of QSM in M1. When all (both positive and negative) QSM values in M1 were considered, only skewness and standard deviation exhibited statistically significant differences between ALS patients and controls, in agreement with the recent findings of Contarino and colleagues [15]. The mean of all QSM values in M1 was the distribution index with the least diagnostic accuracy. This observation corroborates the findings of Contarino and colleagues [15] and can be explained by the fact that increased QSM values in ALS patients’ M1 are confined to only specific portions of the cortex representing the body parts interested by upper motor neuron pathology [14], and involve only the deep cortical layers [1,4,9]. When only the positive QSM values in M1 were considered, all distribution indices (mean value, standard deviation, skewness and kurtosis) exhibited statistically significant differences between the two groups. This observation suggests that a good strategy to highlight differences between ALS patients and controls might be to direct the analysis to positive-only QSM values (as it was carried out also by Schweitzer and colleagues [8]) and to distribution indices that carry information regarding the distribution tails, such as the standard deviation, skewness and kurtosis, whose highly informative roles have been demonstrated also in recent QSM studies on Parkinson’s disease [26] and Multiple System Atrophy [27]. Another important result of this study is the demonstration that improved classification of ALS patients and controls can be achieved by leveraging on the joint use of the distribution indices of QSM values in M1. An SVM classifier that jointly uses all four distribution indices of positive QSM values in M1 allowed to considerably improve the diagnostic accuracy (A = 0.90) with respect to those previously reported [8,28], despite diagnostic accuracies not being able to be directly compared due to differences among patient populations across studies. In the subjects studied here, the diagnostic accuracy based on the radiological assessment of T2* signal hypo-intensity of M1 in susceptibility-weighted imaging [4] yielded a diagnostic accuracy of 0.79.

Considered the low incidence of this disease, the number of ALS patients’ QSM data (51) included in this study should be considered high. However, only a relatively small number of control subjects (10) underwent the same QSM acquisition protocol; therefore, the two groups were numerically unbalanced. Nevertheless, during the training of each SVM classifier, this unbalance was compensated for, and the diagnostic accuracy was cross-validated 1000 times, every time with seven left-out subjects (six patients and one control). Further, as iron concentration increases with age [29], as it has been observed also with QSM [30], it is important to consider age as a possible confounding factor. However, the two subject groups studied here did not significantly differ for age (Mann–Whitney U-test, *p* = 0.15). Moreover, the age-dependent increase in M1 iron concentration is small with respect to inter-subject variability, and, in particular, it is negligible in the age range considered in this study [29]. Taking into account these considerations, the differences in distribution indices of magnetic susceptibility observed in this study should be ascribed to pathology.

These promising results encourage further studies involving also patients with different levels of diagnostic certainty besides probable and definite ALS [16] and ALS mimics, to clarify whether these results can be extrapolated to the whole ALS population, and to corroborate the finding that the joint evaluation of QSM distribution indices could be used as a quantitative biomarker to support the clinical examination in diagnosis and patient monitoring.

## Figures and Tables

**Table 1:** MRI acquisition information. Abbreviations: GRE: Gradient Recalled Echo; SWAN: Susceptibility-Weighted Angiography; A–P: posterior–anterior direction; R–L: right–left direction; I–S: superior–inferior direction; ASSET: Array Coil Spatial Sensitivity Encoding.

Scanner	Discovery MR750 3.0 T
MRI sequence type	3D GRE multi-echo (SWAN)
Time of Repetition (TR) Times of Echo (TE) TE1 : ΔTE : TE16	68.1 ms
	13 ms: 3.4 ms: 64.4 ms
Flip Angle (FA)	15 degrees
Pixel Bandwidth	488.28 Hz
Field of View (FOV)	240 mm (A–P, frequency direction)
	240 mm (R–L, phase encoding)
	120 mm (I–S)
Matrix size	256 × 256 × 120
Phase FOV	0.7
Parallel imaging ASSET factor	2 in phase-encoding direction
Voxel size	0.94 × 0.94 × 1 mm^3^

## Data Availability

Code and data used for statistical analyses may be provided to interested researchers upon request to the corresponding author, after clearance from the IRB.
